# Effects of exercise prior or during pregnancy in high fat diet fed mice alter bone gene expression of female offspring: An experimental study

**Published:** 2017-02

**Authors:** Abbasali Gaeini, Mohamadreza Baghaban Eslaminejad, Siroos Choobineh, Neda Mousavi, Sadegh Satarifard, Leila Shafieineek

**Affiliations:** 1 *Department of Sport Physiology, School of Physical Education and Sport Sciences, University of Tehran, Tehran, Iran*; 2 *Department of Stem Cells and Developmental Biology, Cell Science Research Center, Royan Institute for Stem Cell Biology and Technology, ACECR, Tehran, Iran.*; 3 *Department of Biochemistry and Nutrition, Faculty of Medicine, Zanjan University of Medical Sciences, Zanjan, Iran.*

**Keywords:** Preconception care, Exercise, High-fat diet, Offspring, Bone

## Abstract

**Background:**

Based on different studies it was shown that exercise training is an important factor in preconception and prenatal care.

**Objective:**

The aim of this study was to determine whether regular preconception exercise training with or without exercise training during pregnancy decreases detrimental effects of maternal high fat diet on female offspring bone health.

**Materials and Methods:**

Twenty-four C57BL/6 female mice were fed high-fat diet (35%) and were randomly divided into four groups: trained in preconception period and exercised during pregnancy (TE); trained in preconception periods but unexercised during pregnancy (TC); untrained in preconception periods but exercised during pregnancy (CE); untrained and unexercised (CC). Trained mice were subjected to a protocol of moderate endurance exercise training over a period of 4 weeks before pregnancy. TE and CE Dams groups had access to wheels throughout pregnancy until delivery. Analyses were performed on the female offspring that did not have access to running wheels or exercise training during any portion of their lives. The relative expression levels of β-catenin, Peroxisome proliferator-activated receptor ƴ (PPARƴ), osteoprotegerin (OPG), and Receptor activator of nuclear factor NF-kB ligand (RANKL) were determined by Quantitative RT-PCR (qPCR).

**Results:**

Exercise during pregnancy in isolation had no effect on any measure genes but exercise both before and during pregnancy affected all genes. Exercise only before pregnancy increased β-catenin and OPG and decreased PPARƴ, RANKL, and RANKL/OPG ratio (p<0.001).

**Conclusion:**

This study demonstrated that maternal exercise training before and during pregnancy may modulate the risk of bone disorders in offspring of mothers fed a high-fat diet.

## Introduction

Obesity is widespread throughout the world affecting women of reproductive age and the incidence of maternal obesity is growing at a worrying speed (1). Maternal obesity and consumption high-fat diet (HFD) throughout pregnancy increases the vulnerability of offspring for developing the metabolic disease later in lifespan and so lead to a faulty cycle of trans-generational transmission of disease, suggesting an epigenetic basis (2, 3). 

 The perinatal and preconception periods are now believed to be momentous for the long-term effects on fetal development and may liable offspring to phenotypic alters later in life (4). The Barker hypothesis proposes that the fetal development process is the origin of diseases in offspring, and jeopardized pre-ovulation oocyte quality, mainly related to epigenetic changes, may be vital as well (5).

The evidence is arising that factors in early life (e.g., maternal exercise, diet, and lifestyle or a destructive factor) may persistently alter the postnatal pattern of skeletal growth and consequently influence the risk of osteoporosis in later life (7). Exercise training acting an important role in the growth and preservation of bone mass and strength (8). Bone marrow mesenchymal stem cells have the capability to distinction into several lineages involving osteoblast, adipocyte, and chondrocyte, depending on both extracellular cues and intrinsic signaling pathways (9). 

Peroxisome proliferator-activated receptor ƴ (PPARƴ) curbed osteoblast distinction by switching to adipocyte (10, 11). Receptor activator of nuclear factor NF-kB ligand (RANKL) and osteoprotegerin (OPG) are known as osteoclast differentiation and osteoclastogenesis inhibitory factors, respectively (12, 13). It is showed that PPARƴ controls osteoclast distinction via both performing on hematopoietic cells and exert influence on mesenchymal cells to regulate RANKL/OPG ratio (14). Wnt/β-catenin activation improves osteoblastogenesis and restrains adipogenesis through preventing PPARƴ (15, 16). Both in vivo and in vitro studies have shown that HFD-induced obesity decreases bone mass and quality resultantly activation of PPARƴ and suppression of Wnt/β-catenin (17). 

National Academy of Medicine research (American nonprofit, non-governmental organization) shows a need for studies that advise and encourage pregnant women to modify and election a healthy lifestyle before and throughout pregnancy (18). Healthy lifestyle modifies before and during pregnancy, including appropriate nutrition and regular exercise training, seem to help offspring health and may be facilitated through fluctuations in maternal metabolism, placental growth, and vascularity, oxidative stress, and endothelial cell function (19, 20). Arising evidence reveals that maternal exercise throughout pregnancy may play a vital role in the prevention of chronic diseases in the offspring via fetal programming in utero (21-24).

Progressing studies investigative the association between maternal exercise and offspring health. But, there has been very little research examine the effects of preconception exercise training on offspring, as no information’s are now available on effects of regular preconception exercise training on the bone heath in adult offspring. To our knowledge, the only data existing are those of Rosa *et al*, who examined the effects of moderate exercise throughout pregnancy on bone and body composition of the adult offspring and report that moderate exercise during pregnancy can lead to changes the musculoskeletal system and adiposity in male offspring (25). 

Accordingly, the purpose of current study was to find out whether regular preconception exercise training with or without voluntary exercise throughout pregnancy declines negative effects of maternal HFD on female offspring bone health and which one is most powerful? As maternal obesity may have significant trans-generational impacts, female offspring were considered.

## Materials and methods

**Animal**

**Figure 1:**
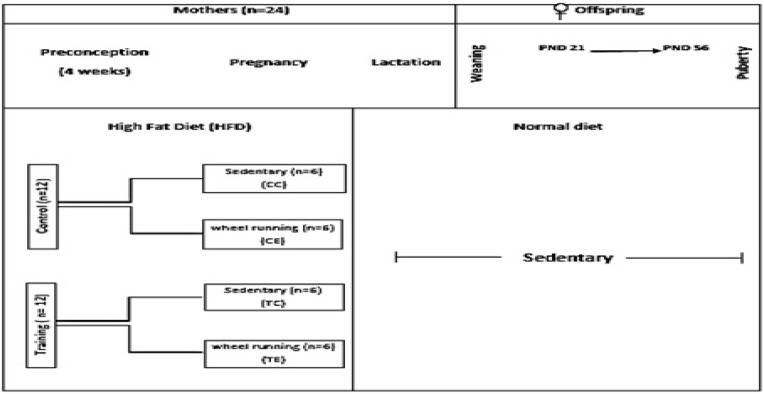
Experimental program. (CC): untrained and unexercised, (CE): untrained in preconception periods but exercised during pregnancy, (TC): trained in preconception periods but unexercised during pregnancy, (TE): trained in preconception period and exercised during pregnancy

Female C57BL/6 mice hold of animal care laboratory of Iran University, Tehran, Iran. After 2 wk of familiarization with a -HFD (35%), twenty-four mice (8 wk old) were randomly divided into four groups (n=6 per group). Each group for 4 wk before and during pregnancy assigned to; trained in preconception period and exercised during pregnancy (TE); trained in preconception periods but unexercised during pregnancy (TC); untrained in preconception periods but exercised during pregnancy (CE); untrained and unexercised (CC) (Figure 1).

The composition of the HFD formulated was based on American Institute of Nutrition-93 (AIN-93) rodent diet composition as recommended by the American Institute of Nutrition with modifications in the fat components to 35% of the total energy from fat (soya oil and lard), by substituting energy from carbohydrate (Table I) (26). 

Diet was prepared at Nutritional Laboratory of the Tehran University of Medical Sciences. At the time of mating, each female mouse and a sedentary male C57BL/6 mouse was held in the cage overnight. Once vaginal plaque approval dams of CE and TE groups were housed individually in cages equipped with running wheels with electronic sensors for counting the round of wheel. Dams had disposed to wheels during pregnancy until delivery. Distance moved was calculated from wheel rotation every 24 hr. The exercise was fully deliberate. We discontinue exercise on the first day of lactation in order to limit our study to the effects of exercise during pregnancy. 

Dams were fed with normal diet (AIN93G). At weaning (Post Natal Day 21^th^) one female offspring was separated from each dam and was housed until 8 wk of age. Offspring were sedentary and were fed with normal diet (AIN93G). Once offspring were 8 wk old (PND56), they were sacrificed by 40 mg/kg ketamine and 8 mg/kg xylasine. The right femur bones were removed from mice and the bones were snap frozen in liquid nitrogen and stored at -80^o^C for extracting the RNA.

**Exercise training protocol**

**Figure 2:**
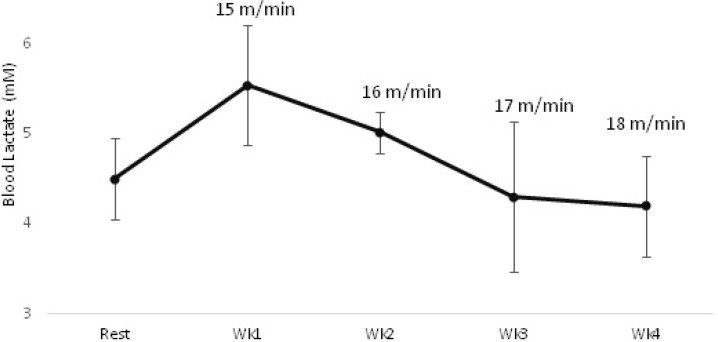
Lactate concentrations in training dams groups

TE and TC dams groups were exercised for 4 weeks, 5 days per week, one session per day. The mice were exposed to a gradual exercise training protocol to make a sure persistent load. After the habituation period for the first week, each session involved a 10 min warm-up at 12 m/min followed by 38.5 min at 15 m/min. This speed approximates below the lactate threshold for untrained C57BL/6 mice (27). During the next 3 weeks, treadmill speed was gradually increased every week, based on the blood lactate levels measured closely after treadmill running a session on a weekly base (Figure 2). In detail, for 2^nd^, 3^rd^, and 4^th^ week the treadmill speed was set at 16 m/min, 17 m/min, and 18 m/min, respectively. For example, the work load for each session during first week was (10 min× 12m/min) + (38.5 min× 15 min/min) = 700 m. 

**Measurement of body weight and food intake during gestation**

During gestation, dams were housed individually, and body and food weights were recorded daily during the experiment by a Marte Scale (EK-3000i) approaching 0.01 g. 

**Gene expression**

The bones were placed in a mortar that was pre-chilled in liquid nitrogen and the bone was pulverized using a pestle until the bone was powdered (28). The powdered bone tissue was transferred to a micro-tube for the addition of the QIAzol Lysis Reagent (1 ml) and was treated with gDNA Eliminator Solution to remove any genomic DNA contamination. Samples were then incubated at room temperature for 15 min after which chloroform was added and vortexed. Then, the samples were centrifuged at 12,000 g for 10 min at 4^o^C and the aqueous phase was transferred to a fresh tube and RNA was extracted and isolated. The RNA was cleaned using an RNeasy® Plus Universal Mini Kit (Qiagen) according to the manufacturer’s protocol. The quality and quantity of isolated RNA were determined using spectrophotometer (WPA Biowave II) measurements followed by storage at -80^o^C prior to further analysis. 

**cDNA synthesis and Quantitative Real-time PCR (qPCR)**

The RNA was converted to cDNA by the PrimeScript^TM^ RT reagent Kit (Perfect Real Time) as per the manufacturer’s instructions. cDNA levels were determined by the StepOne Real-Time PCR system (Applied Biosystems, California, USA). Each 20 µl reaction volume contained 2 µl primers (forward and reverse), 10 µl Power SYBR® Green PCR Master Mix in ABI (Applied Biosystems), 6 µl water and 2 µl of sample cDNA. qPCR analyses for OPG, RANKL, PPARƴ and β-catenin were performed where Glyceraldehyde 3-phosphate dehydrogenase (GAPDH) was used as the housekeeping gene. The amplification profile included one cycle at 95^o^C for 10 min and 40 two-step cycles: 95^o^C for 15 sec and 60^o^C for 60 sec. Primer is presented in table II. The real-time data were analyzed using StepOne software (Applied Biosystems, Life Technologies Corp., Carlsbad CA, USA) to yield relative expression ratios. Quantiﬁcation of mRNA was calculated using the 2^-DDCT^ method as previously described (29).

**Ethical consideration**

This study was an experimental design and carried out in strict accordance with the recommendations in the Guide for the Care and Use of Laboratory Animals of the National Institutes of Health. The study protocol and all animal procedures were approved by the Research Committee of Tehran University, Faculty of Physical Education and Sport Sciences (Permit number: 74/215702). 

**Statistical analysis**

Data were analyzed for significant by One-way analysis of variation followed by Tukey's post hoc test at a p<0.05. Each value was expressed as the mean±SE. 

## Results

**Running wheel distance, dam’s weight, and food intake**

**Figure 3:**
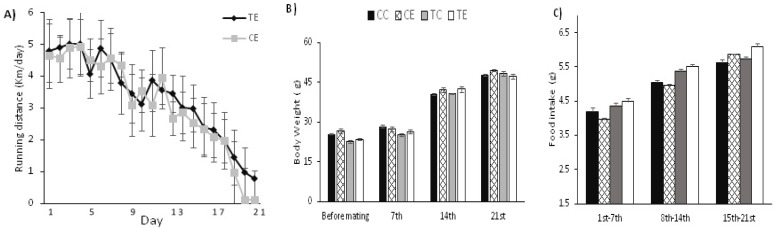
Dams running distance and body weight. The daily voluntary run distances in km are presented in A. The body weights and food intake of pregnant dams in four groups by weeks of pregnancy are shown in B and C. (CC): untrained and unexercised, (CE): untrained in preconception periods but exercised during pregnancy, (TC): trained in preconception periods but unexercised during pregnancy, (TE): trained in preconception period and exercised during pregnancy

There was no significant difference in the distance run between the preconception trained and gestation-only exercised dams in the first (p=0.64), second (p=0.68) and third (p=0.12) weeks of gestation (Figure 3A). Moreover, there were no significant differences in weight gain in the first (p=0.83), second (p=0.43) and third (p=0.28) weeks of gestation and food in the first (p=0.73), second (p=0.22), and third (p=0.52) weeks of gestation (Figure 3B, 3C).

**Gene expression**

**Figure 4:**
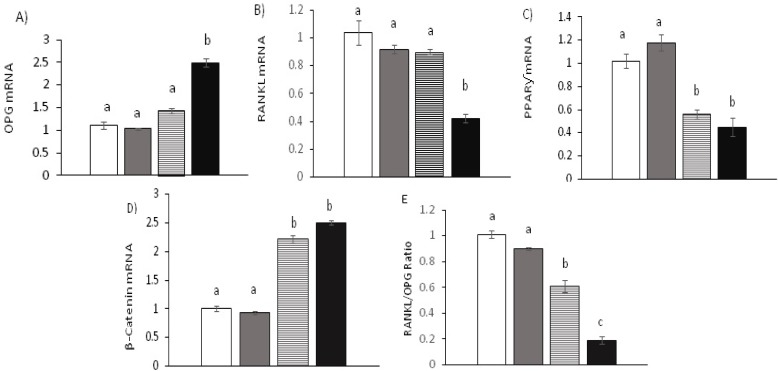
**A, B **and** C; **OPG, RANKL and PPARƴ mRNA expression. **D **and **E; **β-catenin mRNA expression, and RANKL/OPG ratio. Means with different letters differ significantly from each other at p<0.05, a<b<c as determined by one-way ANOVA followed by Tukey’s post hoc analysis for multiple pairwise comparisons. (CC): untrained and unexercised, (CE): untrained in preconception periods but exercised during pregnancy, (TC): trained in preconception periods but unexercised during pregnancy, (TE): trained in preconception period and exercised during pregnancy

OPG, RANKL, β-catenin and PPARƴ mRNA expression are summarized in figure 4. OPG mRNA expression was significantly up-regulated in offspring born to TE dams compared with TC and CE offspring groups (p=0.001) (Panel A, figure 4). In addition, there was no significant difference between CE and TC offspring groups in OPG expression (p=0.73). The fold change level of OPG in the CE offspring group versus the TC offspring were 1.03±0.08 and 1.42±0.06 respectively. RANKL mRNA expression, indicating statistically significant difference induced by structured exercise training in preconception period with voluntary exercise during pregnancy on RANKL fold change (Panel B, figure 4). So that, there were significant differences between TE with TC, and CE offspring groups (p<0.001). However, there was no statistically significant difference between offspring born to CE and TC dams (p=0.99). The fold change level of RANKL in the CE offspring group versus the TC offspring were 0.92±0.03 and 0.9±0.02 respectively. 

PPARƴ mRNA expression, indicating a significant decrease in offspring bone adipogenesis induced by structured exercise training in preconception period (Panel C, figure 4). There were significant differences between CE, and CC compared with TE, and TC offspring groups (p<0.001). However, there were no significant differences between offspring born to TE, and TC dams (p=0.83). The fold change level of PPARƴ in the TC offspring group versus the TE offspring were 0.56±0.04, and 0.45±0.08 respectively. 

β-catenin mRNA expression, highlighting significant up-regulation in offspring born to TC, and TE dams compared with CE, and CC groups (p<0.001) (Panel D, figure 4). Nevertheless, there was no significant difference between β-catenin gene expression levels in TC, and TE offspring groups (p=0.87). The fold change level of β-catenin in the TC offspring group versus the TE offspring was 2.22±0.06, and 2.51±0.08 respectively. Results on RANKL/OPG ratio showed that there were significant differences between CE and CC offspring groups compared with TC and TE groups (p<0.001). There was also a significant difference in RANKL/OPG ratio between offspring born to TC, and TE dams (p<0.001), (Panel D, figure 4). 

**Table I:** Composition of experimental diets (g/kg diet

**Ingredients (g)**	**Experimental diets (AIN93G)**	
	**Normal**	**HFD**
Casein	200	200
L-cystine	3	3
Cornstarch	529	394
Sucrose	100	100
Soy oil	70	70
Lard	0	100
Fiber	50	50
AIN-93 mineral mix	35	35
AIN-93 vitamin mix	10	10
Choline bitartrate	2.5	2.5
Tert-butyl hydroquinone	0.014	0.014
Protein	20	20
Carbohydrate	64	45
Fat	16	35
Total	100	100
Kcal/g	3.9	4.3

AIN: American Institute of Nutrition

HFD: high-fat diet

**Table II:** Primer used in Real-time PCR

**Genes**	**Primers (5’-3’)**	**length (bp, #425)**
*RANKL*	F:5’-CAGCATCGCTCTGTTCCTGTA-3’	21
	R:5’-CTGCGTTTTCATGGAGTCTCA-3’	21
*OPG*	F:5’-GGGCGTTACCTGGAGATCG-3’	19
	R:5’-CGTTGTCATGTGTTGCATTTCC-3’	22
*PPARƴ*	F: 5’- GCCCTTTGGTGACTTTATGGA -3’	21
	R: 5’-GCAGCAGGTTGTCTTGGATG -3’	20
*β-catenin*	F: 5’-CCTCCCAAGTCCTTTATGAATGG-3’	23
	R: 5’-CCGTCAATATCAGCTACTTGCTCTT -3’	25
*GAPDH*	F:5’-GACTTCAACAGCAACTCCCAC -3’	21
	R:5’-TCCACCACCCTGTTGCTGTA -3’	20

RANKL: Receptor activator of nuclear factor NF-kB ligand

OPG: Osteoprotegerin

PPAPƴ: Peroxisome proliferator-activated receptor ƴ

GAPDH: Glyceraldehyde 3-phosphate dehydrogenase

## Discussion

However there has been markedly interest in long-term effects of maternal exercise on separate sides of offspring’s health, the evidence for the impact of regular preconception exercise training on offspring bone health is demanding (30). Both in vivo and in-vitro studies have shown that HFD may be directly accountable for both increased bone marrow adipogenesis and diminished osteoblast differentiation (17). 

The results of this study suggest that well-organized preconception exercise training with voluntary exercise during pregnancy (TE) reduced detrimental effects of maternal HFD on female offspring bone by up-regulated OPG and β-catenin mRNA expression and down-regulated PPARƴ and RANKL gene expressions. Also, we have observed that β-catenin and PPARƴ mRNA expressions significantly up-regulated in female offspring born to dams that had exercise training only in preconception period (TC), while it was down-regulated in untrained in preconception periods but exercised during pregnancy (CE) and untrained and unexercised dams (CC). However, there was no significant difference in β-catenin and PPARƴ expression between TC, and TE offspring groups, highlighting the importance of improving physical fitness in preconception period at least about β-catenin and PPARƴ gene expression. 

It should be noted that bone growth during embryonic development occurs at a very high rate (12). PPARƴ inhibits osteoblast differentiation by shifting towards adipocyte (10, 11). RANKL and OPG was known as osteoclast differentiation and osteoclastogenesis inhibitory factors, respectively (12, 13). It is described that PPARƴ controls osteoclast distinction via both performing on hematopoietic cells and influencing mesenchymal cells to regulator RANKL/OPG ratio (14). Wnt/β-catenin activation enhances osteoblastogenesis and suppresses adipogenesis by inhibiting PPARƴ (15, 16).

We speculated that there are some possible mechanisms responsible for these findings; one is that the preconception exercise training of dams may be indirectly and through decreased serum levels of free fatty acids in pregnant dams not only activate β-catenin to stimulate osteogenesis, but also suppress PPARƴ to inhibit adipogenesis. Another possible mechanism is that preconception exercise training may be directly led to inhibition of the decrease β-catenin mRNA expression that induced by HFD in dams and subsequent suppress PPARƴ mRNA expression. One study demonstrated that higher levels of non-esterified free fatty acids (NEFA) in serum from rats made obese by HFD-feeding activate PPARƴ and suppress β-catenin mRNA expression and impaired bone formation due to stimulation of bone marrow adipogenesis. 

endogenous β-catenin silenced in the pre-osteoblast cause to over-expression of PPARƴ, exhibiting this gene acts as a decreasing of PPARƴ in the osteoblast distinction (17). Regarding osteoclast differentiation one of the most important factors is the cytokine RANKL. The binding of RANKL to its receptor RANK can be physiologically reserved by its receptor OPG, whose in-activating elements excessive bone resorption (31). Our results indicated that structured preconception exercise training with voluntary exercise during pregnancy led to significantly up and down regulated of OPG and RANKL mRNA expression respectively, in female offspring bone. However, this finding was not observed in offspring born to dams only exercise in preconception period. In the other words, preconception exercise training without voluntary exercise during pregnancy could not inhibit OPG mRNA down-regulation induced by HFD in offspring bone. 

In the current study, maternal preconception exercise training was absolutely restrained, therefore physical fitness in groups equivalently increased, implying that along with improving physical fitness before pregnancy, conservation of it is essential. However, RANKL/OPG ratio improved in offspring of TE, and TC dams groups. presently, there is no data available on the effects of exercise training in pre and during pregnancy related to this topic, but there is a substantial body of evidence that exercise training affects bone metabolism, growth, remodeling, and turnover (32). These processes are under the control of several endogenous factors, such as hormones, growth factors and cytokines, and exogenous factors, such as mechanical loading (33). 

In vitro and in vivo studies have shown that mechanical stimulant can prohibit osteoclast formation and activity by altering the RANKL/OPG ratio in favor of OPG (34). It is reported that exercise training has time-specific effects on fetal and placental growth and fetoplacental compatibility are dependent on the period of pregnancy in which exercise training begins and preserved (35). Clapp and colleagues described the time-dependent effects of exercise during pregnancy (36). They examined the effects of vigorous weight-bearing aerobic exercise in physically fit women whereas exercise volume was changed in mid-pregnancy. Mid-trimester placental growth rates considerably were better in those women who took part in a moderate- or high-volume exercise program during early pregnancy in comparison with a group who reduced their exercise volumes to a low level during early pregnancy. Accordingly, we guess that time-dependent effects of exercise training can be lengthy from preconception period up to delivery and both oocytes, and embryos are vulnerable to factors of maternal lifestyle. Of this, the creation and advancement of oocytes rely on the follicular milieu, which can be modified or compromise by the mother’s health and lifestyle (37).

Taken together, this study suggests that a sedentary lifestyle including either physical inactivity or a high-fat diet can result in a ‘bad epigenetic phenotype’ and can be transmitted to the next generation. The preconception period is an “important time” and represents a unique opportunity to improve women health and physical fitness before conception (38). It is essential to note that pregnancy is not a time for greatly improving physical fitness. Exercise training has positive effects on woman’s bone health and this also transmitted to her offspring. Although, obesity and over nutrition in individuals of reproductive age can propagate risk to subsequent generations via nongenetic or epigenetic factors (35, 39).

## Conclusion

Our findings suggest that maternal physical fitness level and exercise during pregnancy may modify destructive effects of maternal high fat diet to potentiate protection from the risk of offspring bone disorders in later life, and reveal a novel link between maternal physical fitness level, and offspring health. However, further studies examining both mRNA and protein expression, as well as epigenetic changes such DNA methylation will provide more insight into specific factors, and pathways underlying the effects of preconception exercise training on offspring’s bone health. 

## Note

***This article extracted from Ph.D. thesis. (Leila Shafieneek)***
